# Fatty Acid and Hopanoid Adaption to Cold in the Methanotroph *Methylovulum psychrotolerans*

**DOI:** 10.3389/fmicb.2019.00589

**Published:** 2019-04-05

**Authors:** Nicole J. Bale, W. Irene C. Rijpstra, Diana X. Sahonero-Canavesi, Igor Y. Oshkin, Svetlana E. Belova, Svetlana N. Dedysh, Jaap S. Sinninghe Damsté

**Affiliations:** ^1^Department of Marine Microbiology and Biogeochemistry, NIOZ Royal Institute for Sea Research, and Utrecht University, Texel, Netherlands; ^2^Research Center of Biotechnology of the Russian Academy of Sciences, Winogradsky Institute of Microbiology, Moscow, Russia; ^3^Department of Earth Sciences, Faculty of Geosciences, Utrecht University, Utrecht, Netherlands

**Keywords:** methanotroph, bacteria, methane, temperature, fatty acid, hopanol

## Abstract

Three strains of aerobic psychrotolerant methanotrophic bacteria *Methylovulum psychrotolerans*, isolated from geographically remote low-temperature environments in Northern Russia, were grown at three different growth temperatures, 20, 10 and 4°C and were found to be capable of oxidizing methane at all temperatures. The three *M. psychrotolerans* strains adapted their membranes to decreasing growth temperature by increasing the percent of unsaturated fatty acid (FAs), both for the bulk and intact polar lipid (IPL)-bound FAs. Furthermore, the ratio of βOH-C_16:0_ to *n*-C_16:0_ increased as growth temperature decreased. The IPL head group composition did not change as an adaption to temperature. The most notable hopanoid temperature adaptation of *M. psychrotolerans* was an increase in unsaturated hopanols with decreasing temperature. As the growth temperature decreased from 20 to 4°C, the percent of unsaturated *M. psychrotolerans* bulk-FAs increased from 79 to 89 % while the total percent of unsaturated hopanoids increased from 27 to 49 %. While increased FA unsaturation in response to decreased temperature is a commonly observed response in order to maintain the liquid-crystalline character of bacterial membranes, hopanoid unsaturation upon cold exposition has not previously been described. In order to investigate the mechanisms of both FA and hopanoid cold-adaption in *M. psychrotolerans* we identified genes in the genome of *M. psychrotolerans* that potentially code for FA and hopanoid desaturases. The unsaturation of hopanoids represents a novel membrane adaption to maintain homeostasis upon cold adaptation.

## Introduction

*Methylovulum psychrotolerans* is a recently described species of aerobic methanotrophic bacteria, isolated from several low-temperature habitats in Northern Russia, such as cold methane seeps and subarctic freshwater lake sediments (Oshkin et al., 2016). These psychrotolerant methanotrophs are able to grow at temperatures between 2°C and 36°C and, although their optimum temperature range is 20–25°C, they also grow well at lower temperatures, down to 4°C. Members of the genus *Methylovulum* are characterized as Type I methanotrophs, as they belong to the *Gammaproteobacteria* and assimilate C1 compounds via the ribulose monophosphate pathway (RuMP) (Hanson and Hanson, 1996; Trotsenko and Murrell, 2008). The lipid characteristics of Type I methanotrophs include high levels of *n*-C_16_ fatty acids (C_16_ FAs), and in particular, unsaturated *n*-C_16_ FAs (Hanson and Hanson, 1996; Nichols et al., 1985). However, species from the genera *Methylovulum* have been reported to also contain high levels of saturated *n*-C_16_ FA and *n*-C_14_ FA (Iguchi et al., 2011; Oshkin et al., 2016). β-hydroxy FAs have been detected in a range of methanotrophic bacteria (Bowman et al., 1991) and their source has been identified as the lipid A component of lipopolysaccharides (Wollenweber and Rietschel, 1990), which are a major constituent of the outer membrane of Gram-negative bacteria (Keinänen et al., 2003; Wollenweber and Rietschel, 1990). Methanotrophs also produce bacteriohopanepolyols (BHPs) which can be tetra, penta or hexafunctionalised (Cvejic et al., 2000a; van Winden et al., 2012b; Rush et al., 2016; Osborne et al., 2017). BHPs have been identified with a range of modifications including 3β-methylation and unsaturations at the Δ^6^ and Δ^11^ positions (Talbot et al., 2007a). BHPs (and hopanoid alcohols derived thereof) with an unsaturation at the Δ^11^ position have been observed in a *Methylovulum*-like strain M200 (van Winden et al., 2012b), and have been observed in combination with a 3β methylation in the thermophilic *Methylocaldum szegediense* (Cvejic et al., 2000a) and in a range of acetic acid bacteria (Rohmer and Ourisson, 1976; Simonin et al., 1994; Herrmann et al., 1996).

The effect of temperature on bacterial membrane lipids has been extensively studied (Suutari and Laakso, 1994; Chintalapati et al., 2004; Barria et al., 2013). Indeed, in order to maintain sufficient membrane fluidity at low temperatures, bacteria adapt their membranes to lower the phase-transition temperature below which their membrane changes from a “fluid” (liquid-crystalline) to a “rigid” phase (Chattopadhyay, 2006; Guschina and Harwood, 2006; Shivaji and Prakash, 2010; Siliakus et al., 2017). These membrane adaptations to cold include increases in unsaturated FAs, short chain FAs and branched chain FAs as well as the incorporation of carotenoids and glycolipids. Several studies have described the membrane lipid composition and adaptation to cold in psychrophilic bacteria (which have an optimum growth temperature of < 15°C) including *Clostridium psychrophilum*, *Colwellia psychrerythraea* and *Psychromonas ingrahamii* (Breezee et al., 2004; Auman et al., 2006; Guan et al., 2013; Wan et al., 2016). Membrane adaptation to cold in psychrotolerant bacteria (which have an optimum temperature between 20 and 40°C, but are also capable of growth around 0°C) has been studied for species including *Sphingobacterium antarcticus* and *Micrococcus roseus* (Chattopadhyay et al., 1997; Jagannadham et al., 2000). A range of species-specific adaptions were described for these psychrotolerant microorganisms including increases in unsaturated FAs, both increases and decreases in branched chain FAs and incorporation of polar and non-polar carotenoids into the membrane.

Changes in hopanoids with changing growth temperature has been reported for non-methanotrophs, such as *Frateuria aurantia* (Joyeux et al., 2004), *Zymomonas mobilis* (Hermans et al., 1991) and *Bacillus acidocaldarius* (Poralla et al., 1984). However, only a limited number of studies have examined the effect of temperature on the lipid composition of methanotrophic bacteria: a study of “CEL 1923” [thought to be *Methylomonas methanica*; Jahnke et al. (1999)], a mesocosm experiment with *Sphagnum* moss colonized by symbiotic methanotrophs (van Winden, 2011; van Winden et al., 2012a) and a study of methane-amended aerobic river-sediment incubations (Osborne et al., 2017).

In this study, three strains of *M. psychrotolerans* (Sph1^T^_,_ OZ2, and Sph56) were grown at three different growth temperatures, 20, 10 and 4°C. In order to examine growth temperature-driven changes in their lipid distribution, the FA distribution (bulk and IPL-bound) was examined at each growth temperature. The changes in hopanoids were examined by analysis of the hopanols formed by Rohmer degradation of intact BHPs.

## Materials and Methods

### Strains and Culture Conditions

**FIGURE 1:**
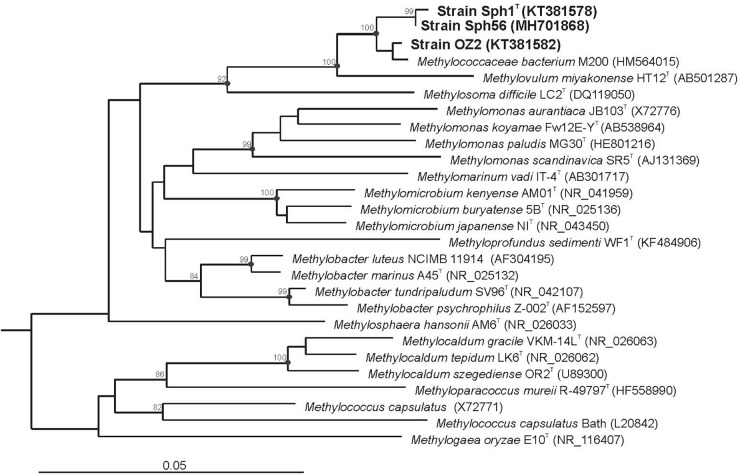
16S rRNA gene based neighbor-joining tree showing the phylogenetic position of strains Sph1^T^, OZ2 and Sph56 in relation to other members of the family *Methylococcaceae*. Bootstrap values (percentages of 1000 data resamplings) > 80% are shown. Black circles indicate that the corresponding nodes were also recovered in the maximum-likelihood and maximum- parsimony trees. The type II methanotrophs *Methyloferula stellata* AR4 (FR686343), *Methylocella silvestris* BL2 (AJ491847), *Methylocapsa acidiphila* B2 (AJ278726), *Methylosinus sporium* NCIMB 11126 (Y18946), *Methylosinus trichosporium* OB3b (Y18947) and *Methylocystis parvus* OBBP (Y18945) were used as an outgroup. Bar, 0.05 substitutions per nucleotide position.

Three strains of *Methylovulum psychrotolerans* isolated from different geographical locations in Northern Russia were used in this study. The type strain of this species, Sph1^T^, was obtained from a cold methane seep located in the valley of the river Mukhrinskaya, Irtysh basin, West Siberia (60°53.358′ N 68°42.486′ E). Strain OZ2 was isolated from sediments of a subarctic, shallow, unnamed freshwater lake in Archangelsk region (67° 36.567′ N 53°35.317′ E). These two strains were characterized in detail by Oshkin et al. (2016). The third strain, designated Sph56, was isolated from sediments of an arctic, unnamed freshwater lake located on the island Belyy in the Kara Sea (73°20.25′ N 70°10.59′ E) (Supplementary Figure S1). Cell morphology and physiological characteristics of strain Sph56 were highly similar to those of strains Sph1^T^ and OZ2. In addition, the 16S rRNA gene sequence determined for strain Sph56 (GenBank Accession number MH701868) displayed 99.5 % identity to the corresponding gene sequences from strains Sph1^T^ and OZ2 (Figure 1). Based on these data, strain Sph56 has also been identified as belonging to the species *Methylovulum psychrotolerans*.

For lipid analysis, the three strains were cultivated in NMS medium containing (in gram per liter) MgSO_4_, 1; KNO_3_, 1; CaCl_2_, 100; KH_2_PO_4_, 0.272; Na_2_HPO_4_× 12H_2_O, 0.717 with the addition of 0.1 % (v/v) of a trace elements stock solution containing (in grams per liter) EDTA, 5; FeSO_4_.7H_2_O, 2; ZnSO_4_.7H2O, 0.1; MnCl_2_.4H_2_O, 0.03; CoCl_2_.6H_2_O, 0.2; CuCl_2_.5H_2_O, 0.1; NiCl_2_.6H_2_O, 0.02; and Na_2_MoO_4_, 0.03. The medium pH was 6.8. The flasks of a total volume 500 ml were filled to 20 % capacity with NMS medium, sealed with rubber septa, and CH_4_ (30%, v/v) was added to the headspace using syringes equipped with disposable filters (0.22 μm). The cultures were grown in a Multitron Pro shaker incubator (Infors HT, Switzerland) at 4, 10 and 20°C, respectively, and were harvested in late exponential growth phase.

### Measurements of Methane Oxidation Rates

Prior to kinetic experiments, strains Sph1^T^, OZ2 and Sph56 were maintained in NMS medium at 4, 10 and 20°C, respectively, for one month, with regular transfers after 5–7 days. Methane oxidation rates were determined as described by Knief and Dunfield (2005). Briefly, the cells were counted, and the cultures were diluted to 1 × 10^8^ cells ml^−1^ with 0.5 mM phosphate buffer. Chloramphenicol (1.25 mg l^−1^) was added to the experimental flasks to inhibit further cell growth. Aliquots (10 ml) of cell suspensions were added to 500-ml flasks, which were sealed gas-tight with butyl rubber septa. Methane was added to final mixing ratios of 0.5% (v/v). The flasks were fixed in a horizontal position on a rotary shaker with the temperature settings of 4, 10 and 20°C, respectively, and shaken at 110 rpm. The decrease of methane concentration in the headspace was followed over time using a gas chromatograph (Chromatec crystal 5000, Chromatec, Russia) equipped with a flame ionization detector. Methane oxidation rates were estimated by linear regressions of CH_4_ concentrations versus time.

### Fatty Acid Analysis

For (bulk) fatty acid (FA) analysis, aliquots of the lyophilized cells from duplicate cultures were hydrolyzed by refluxing with 1.5N HCl:MeOH solution for 3 h. After adjustment of the pH to 4–5 with a 2 N KOH/MeOH (1/1, v/v) solution, extraction with dichloromethane (DCM) was carried out. The resulting extract was methylated with diazomethane in diethyl ether which was removed under a stream of N_2_. Before analysis an aliquot was treated with BSTFA in pyridine to derivatize alcohol groups and then brought to a final volume with ethyl acetate. FA methyl ester (FAME) identification was carried out using gas chromatography-mass spectrometry (Thermo Finnigan TRACE GC-MS). FAMEs were separated using a CP-SIL 5CB capillary column (length 25 m × internal diameter 0.32 mm, coating 0.12 μm) with the following oven conditions: initial temperature 70°C, increasing to 130°C by 20°C min^−1^, then increasing to 320°C by 4°C min^−1^ and held for 10 min. MS operating parameters were: electron multiplier 1663 V; source temperature 250°C; full scan *m/z* 50–800; scan time 0.33 s. MS data were acquired and processed using the Thermo Finnigan Xcalibur software. FAMEs were identified based on literature data and library mass spectra. Double bond positions were determined, where possible, using dimethyldisulfide (DMDS) derivatization of the FAMEs. To this end, extracts were derivatized in hexane (100 μl) with DMDS (Merck ≥ 99%; 100 μl) and I_2_/ether (60 mg ml^−1^; 20 μl) and heated overnight at 40°C. Hexane (400 μl) was then added with Na_2_S_2_O_3_ (5% aqueous solution; 200 μl) to deactivate the iodine. The hexane layer was removed, and the aqueous phase washed with hexane (× 2). The hexane layers were combined and analyzed by GC-MS as described above.

### Hopanoid Analysis

For the detection of the presence of biohopanoids, lyophilized cells were directly treated with periodic acid/sodium borohydride to convert bacteriohopanepolyols (BHPs) into GC-amenable hopanoid alcohols (hopanols) following procedure 2 described by Rohmer et al. (1984) with some modifications. Lyophilized cells (ca. 10 mg) were stirred with 1 ml of a solution of periodic acid (30 mg) in tetrahydrofuran/water (8:1, v/v) at room temperature for 1 h. After addition of water (1 ml), the lipids were extracted three times with DCM (2 ml), and the solution was dried over anhydrous Na_2_SO_4_, and evaporated to dryness. The residue was treated with 20 mg of NaBH_4_ in 1 ml methanol by stirring at room temperature for 1 h. After addition of a solution of KH_2_PO_4_ (1 ml, 200 mM), the hopanols were extracted with DCM. The obtained reaction mixture was methylated with diazomethane and separated over a small column with activated Al_2_O_3_ into an apolar and a polar fraction using DCM and DCM-MeOH (2:1, v/v) as eluent, respectively. The polar fractions were silylated with N,O-bis(trimethylsilyl)-fluoroacetamide in pyridine at 60°C for 20 min and analyzed by GC and GC-MS. The distribution of hopanols was obtained by integration of the appropriate peaks. GC and GC-MS analysis was performed as described above for fatty acid analysis.

### Intact Polar Lipid Analysis

The lyophilized biomass of Sph56 (single replicates from 20, 10 and 4°C) was extracted using a modified Bligh-Dyer procedure (Bligh and Dyer, 1959). Briefly, the biomass was extracted ultrasonically three times for 10 min in a solvent mixture of methanol:dichloromethane:phosphate buffer (2:1:0.8, v:v). After sonication, the combined supernatants were phase-separated by adding additional dichloromethane and buffer to a final solvent ratio of 1:1:0.9 (v:v). The organic phase containing the IPLs was collected and the aqueous phase re-extracted three times with dichloromethane. Finally, the combined extract was dried under a stream of N_2_ gas. Before analysis the extract was redissolved in a mixture of MeOH:DCM (9:1, v:v) and aliquots were filtered through 0.45 μm regenerated cellulose syringe filters (4 mm diameter; Grace Alltech, Deerfield, IL, United States). Analysis of extracts was carried out using an Ultra High Pressure Liquid Chromatography-High Resolution Mass Spectrometry (UHPLC-HRMS) according to the reversed phase method of Wörmer et al. (2013) with some modifications. We used an Ultimate 3000 RS UHPLC, equipped with thermostatted auto-injector and column oven, coupled to a Q Exactive Orbitrap MS with Ion Max source with heated electrospray ionization (HESI) probe (Thermo Fisher Scientific, Waltham, MA, United States). Separation was achieved on an Acquity BEH C18 column (Waters, 2.1 × 150 mm, 1.7 μm) maintained at 30°C. The eluent composition was (A) MeOH:H_2_O:formic acid:14.8 M NH_3aq_ (85:15:0.12:0.04 [v:v:v:v]) and (B) IPA:MeOH:formic acid:14.8 M NH_3aq_ (50:50:0.12:0.04 [v:v:v:v]). The elution program was: 95% A for 3 min, followed by a linear gradient to 40% A at 12 min and then to 0% A at 50 min, this was maintained until 80 min. The flow rate was 0.2 mL min^−1^. Positive ion ESI settings were: capillary temperature, 300°C; sheath gas (N_2_) pressure, 40 arbitrary units (AU); auxiliary gas (N_2_) pressure, 10 AU; spray voltage, 4.5 kV; probe heater temperature, 50°C; S-lens 70 V. Target lipids were analyzed with a mass range of m/z 350–2000 (resolving power 70,000 ppm at *m/z* 200), followed by data-dependent tandem MS^2^ (resolving power 17,500 ppm), in which the ten most abundant masses in the mass spectrum were fragmented successively (stepped normalized collision energy 15, 22.5, 30; isolation width, 1.0 *m/z*). The Q Exactive was calibrated within a mass accuracy range of 1 ppm using the Thermo Scientific Pierce LTQ Velos ESI Positive Ion Calibration Solution. During analysis dynamic exclusion was used to temporarily exclude masses (for 6 s) in order to allow selection of less abundant ions for MS^2^. The relative abundance of peak areas does not necessarily reflect the actual relative abundance of the different compounds, however, this method allows for comparison between the samples analyzed in this study. The IPL groups were identified through comparison with fragmentation patterns of authentic standards, as described in Brandsma et al. (2012). The chain length and number of double bond equivalents of the IPL-bound fatty acids (FA) were determined by either the fragment ions or neutral losses diagnostic for FAs obtained in the MS^2^ spectra (Brügger et al., 1997; Brandsma et al., 2012). The peak areas were determined from extracted ion chromatograms of the dominant ion formed for each individual IPL species.

### Genomic Analyses

The sequences of putative fatty acid desaturase (POZ50891.1) and sterol desaturase (POZ53527.1) of *M. psychrotolerans* Sph1^T^ (GenBank accession no. PGFZ01000000; Oshkin et al., 2018) were identified with the PSI-BLAST algorithm using the (5) fatty acid desaturase (5-Des; O34653) from *Bacillus subtilis* and sterol desaturase ERG3 (P32353) from *Saccharomyces cerevisiae* as queries. The sequences were aligned with MAFFT (Katoh and Standley, 2013) in the http://www.ebi.ac.uk/Tools server (Chojnacki et al., 2017) using the BLOSUM62 substitution matrix with a gap open penalty of 1 and a gap extension penalty of 0.05. The alignment was edited with Jalview (Waterhouse et al., 2009) and the presence of conserved histidine rich motifs was verified.

## Results and Discussion

### Methane Oxidation at Different Temperatures

**FIGURE 2:**
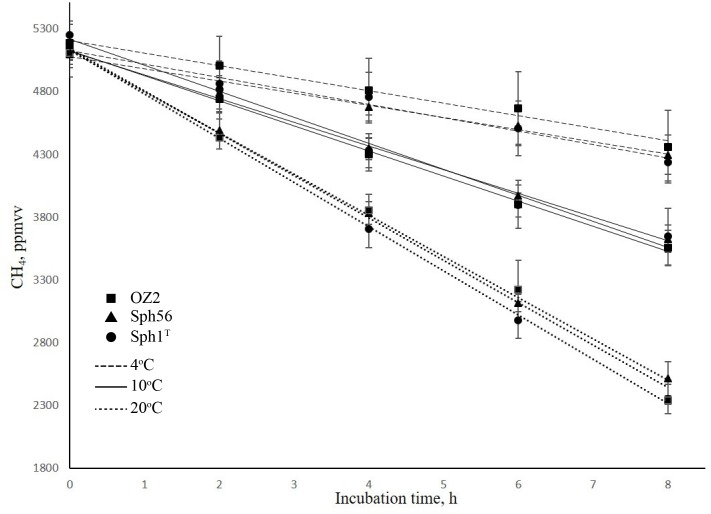
Dynamics of methane concentration in the experimental flasks with strains Sph1^T^ (circles), OZ2 (squares), and Sph56 (triangles) at different incubation temperatures. Dotted, solid and dashed lines indicate incubations at 20, 10 and 4°C, respectively. The linear decline in methane concentration indicates that cell growth did not occur during the incubation period. Data are means of triplicate.

The decline of CH_4_ concentration in the experimental flasks incubated at different temperatures is shown in Figure 2. The highest methane oxidizing activities (6.9–7.4 × 10^−15^mol CH_4_ h^−1^ cell^−1^) were observed at 20°C, which is consistent with the original description of *Methylovulum psychrotolerans* as showing growth optimum at 20–25°C (Oshkin et al., 2016). Methane oxidation rates measured for these methanotrophs at 10°C were in the range 3.9–4.4 × 10^−15^mol CH_4_ h^−1^ cell^−1^, thus constituting 57–60% of the corresponding activities at 20°C. As a rule, lowering the temperature by 10°C induces a two- to fourfold decrease in enzyme activity (i.e., the so-called Q10 value; Feller and Gerday, 2003). Methanotrophs examined in our study, therefore, were relatively resistant to decrease in 10°C away from their temperature optimum. The methane oxidation rates determined at 4°C were in the range 2.0–2.3 × 10^−15^mol CH_4_ h^−1^ cell^−1^, which is twice lower as those at 10°C (Table 1). All three strains of the species *Methylovulum psychrotolerans*, therefore, could be regarded as psychrotolerant mesophiles capable of oxidizing methane at low temperatures, down to 4°C. The growth rates, doubling times and the growth dynamics of the three strains at the three temperatures are given in Supplementary Tables S1, S2 and Figure S2.

**Table 1:** Methane oxidation rates ( × 10^−15^ mol CH_4_ h^−1^ cell^−^1) of *Methylovulum psychrotolerans* strains Sph1^T^, OZ2, and Sph56 measured at 4, 10 and 20°C and calculated per cell.

Strain	Temperature (°C)		
	20	10	4
Sph1^T^	7.40 ± 0.39	4.40 ± 0.36	2.30 ± 0.17
OZ2	7.10 ± 0.35	4.20 ± 0.34	2.10 ± 0.31
Sph56	6.90 ± 0.24	3.90 ± 0.23	2.00 ± 0.26

### The Lipid Distribution in *Methylovulum psychrotolerans*

The three *M. psychrotolerans* strains examined in duplicate contained nine bulk fatty acids (FAs): C_14:1ω7_, C_14:0_, C_15:0_, C_16:1ω8c_, C_16:1ω7c_, C_16:1ω6c_, C_16:1ω5tr_, C_16:0_, βOH-C_16:0_. C_16:1ω8c_ was previously considered unique to type I methanotrophs (Sundh et al., 2005; Bowman, 2006) but is now known to also be produced by type II methanotrophs (Dedysh et al., 2007; Bodelier et al., 2009). However C_16:1ω8c_ has been reported as the dominant FA in *Methylomonas methanica*, *Methylomonas fodinarum* and *Methylomonas aurantiaca* (Bowman et al., 1991). C_16:1ω7c_ is a more cosmopolitan bacterial FA, not specific to methanotrophs. Another species of the genus *Methylovulum*, *Methylovulum miyakonense*, has been reported to have a similar FA distribution to that of *M. psychrotolerans* (Oshkin et al., 2016).

Three Bligh-Dyer extracts of one strain (Sph56), grown at 20, 10 and 4°C, were examined for their intact polar lipid (IPL) distribution. The IPLs detected had either phosphatidylglycerol (PG) or phosphatidylethanolamine (PE) head groups, with a range of FA combinations (Table 2). The head group composition was very similar to that of a range of methanotrophic bacteria described previously (Fang et al., 2000). Additionally, a lyso-PE, (PE in which one of the FA chains is not present) was detected. The majority of IPLs were PE, with the ratio of PG/PE as 0.2. Where possible, the chain length and number of double bond equivalents of the IPL-bound FAs were determined by either the fragment ions diagnostic for FAs obtained in the MS^2^ (Brügger et al., 1997; Brandsma et al., 2012). Where this was not possible (cf. Table 2) an informed estimation of the FAs associated with each IPL was made. In addition to the C_14:0_, C_14:1_, C_15:0_, C_16:0_ and C_16:1_ detected in the bulk FAs, C_15:1_ and C_16:2_ were also detected. The C_15:1_ and C_16:2_ were not detected in the bulk FAs, probably due to their very low abundance and differences in the limit of detection for the different analytical methods applied.

**Table 2:** Relative percentage of intact polar lipids in *Methylovulum psychrotolerans* (strain Sph56) measured at 4, 10 and 20°C.

		20°C	10°C	4°C
IPLs	Lyso-PE 16:1^∗^	1.2	4.3	2.2
	PE 28:0 (14:0,14:0)^∗^	1.4	1.0	0.8
	PE 28:1 (14:0,14:1)	2.3	6.7	6.3
	PE 28:2 (14:1,14:1)^∗^	0.0	0.3	0.3
	PE 29:0 (14:0, 15:0)^∗^	0.2	0.3	0.2
	PE 29:1 (14:1, 15:0)^∗^	0.3	1.2	1.3
	PE 30:0 (14:0, 16:0)^∗^	2.2	0.5	0.4
	PE 30:1 (14:0, 16:1)^∗^	14	14	13
	PE 30:2 (14:1, 16:1)^∗^	3.6	14	16
	PE 31:0 (15:0, 16:0)^∗^	0.3	0.0	0.0
	PE 31:1 (15:0, 16:1)^∗^	2.5	2.6	3.0
	PE 31:2 (15:1, 16:1)^∗^	0.5	1.6	2.0
	PE 32:0 (16:0, 16:0)^∗^	2.2	0.1	0.1
	PE 32:1 (16:0, 16:1)^∗^	21	5.8	5.8
	PE 32:2 (16:1, 16:1)^∗^	33	33	33
	PE 32:3 (16:1, 16:2)^∗^	0.0	0.1	0.1
	PG 28:0 (14:0,14:0)^∗^	0.2	0.2	0.1
	PG 28:1 (14:0,14:1)	0.2	0.8	0.8
	PG 29:1 (14:1, 15:0)	0.0	0.1	0.1
	PG 30:0 (14:0, 16:0)^∗^	0.5	0.1	0.1
	PG 30:1 (14:0, 16:1)^∗^	2.5	3.3	2.9
	PG 30:2 (14:1, 16:1)^∗^	0.2	2.0	2.4
	PG 31:1 (15:0, 16:1)^∗^	0.2	0.1	0.2
	PG 31:2 (15:1, 16:1)^∗^	0.0	0.1	0.1
	PG 32:0 (16:0, 16:0)^∗^	0.4	0.0	0.0
	PG 32:1 (16:0, 16:1)^∗^	4.3	0.8	0.9
	PG 32:2 (16:1, 16:1)^∗^	6.8	7.2	8.1
	PG/PE ratio	0.2	0.2	0.2
IPL-bound FAs	Total C_16_	82	71	70
	Total C_15_	2.0	3.0	3.5
	Total C_14_	16	26	26
	Total unsaturated	69	80	81

*^∗^IPL-bound FA combination confirmed by MS^2^ diagnostic fragment ions.*

**FIGURE 3:**
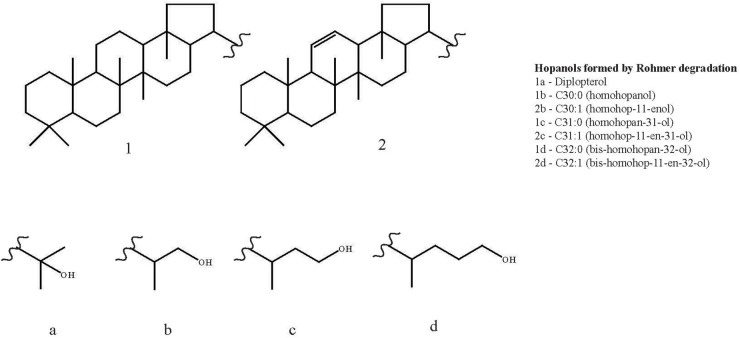
Structures of diplopterol and the hopanols formed by Rohmer degradation of bacteriohopanepolyols (BHPs) identified across the three *Methylovulum psychrotolerans* strains: C_30:0_ (homohopanol), C_30:1_ (homohop-11-enol), C_31:0_ (homohopan-31-ol), C_31:1_ (homohop-11-en-31-ol), C_32:0_ (*bis*-homohopan-32-ol) and C_32:1_ (*bis*-homohop-11-en-32-ol).

Six hopanols formed by Rohmer degradation of BHPs were identified across the duplicates of the three strains (see Figure 3 for structures): C_30:0_ (homohopanol), C_30:1_ (homohop-11-enol), C_31:0_ (homohopan-31-ol), C_31:1_ (homohop-11-en-31-ol), C_32:0_ (*bis*-homohopan-32-ol) and C_32:1_ (*bis*-homohop-11-en-32-ol). The double bond position in the three unsaturated hopanols was confirmed as Δ^11^ by both the absence of *m/z* 119 ion peaks and the presence of M - 192 Da ion peaks in their EI mass spectra (Cvejic et al., 2000a,b; Talbot et al., 2007b; van Winden et al., 2012b). These hopanols are the product of bacteriohopanepolyols (BHPs): tetrafunctionalised BHPs give C_32_ hopanols, pentafunctionalised BHPs give C_31_ hopanols and hexafunctionalised BHPs give C_30_ hopanols. Diplopterol, not formed by degradation of a BHP, was also detected in the three strains, although not in the cultures grown at 20°C. The ratio of diplopterol to the sum of the six hopanols formed by Rohmer degradation of BHPs was on average at 0.1 ± 0.0 at both 10 and 4°C (Table 3). The three strains exhibited similar hopanol distributions to each other (see Table 3 for data for the individual strains), although to a lesser degree than with the FAs. Tetra, penta and hexafunctionalised BHPs, from which the hopanols analyzed in this studied are derived, have been described across a range of methanotrophs, including from the genera *Methylomarinum*, *Methylomarinovum*, *Methylomicrobium*, (Rush et al., 2016), *Methylomonas*, *Methylosinus*, *Methylocella*, *Methylacidiphilium* (van Winden et al., 2012b), *Methylobacter* (Osborne et al., 2017) as well as in a *Methylovulum*-like strain M200 (van Winden et al., 2012b). Unsaturated aminotriol and aminotetrol BHPs have only to date been described in a *Methylovulum*-like strain M200 (van Winden et al., 2012b) which suggests that they are characteristic of the *Methylovulum* genera. The unsaturated aminopentol BHP has been described in the *Methylovulum*-like strain M200 and a *Methylomonas*-like strain (van Winden et al., 2012b) as well as in three strains of *Methylobacter* (Osborne et al., 2017). Additionally, the thermophilic *Methylocaldum szegediense* was found to contain a Δ^11^ unsaturated aminopentol BHP in combination with a methyl group at the 3β position (Cvejic et al., 2000a). Hopanoids with Δ^11^ unsaturations have also been identified in a range of acetic acid bacteria (Rohmer and Ourisson, 1976; Simonin et al., 1994; Herrmann et al., 1996).

**Table 3:** Relative percentage of hopanols (not including diplopterol) formed by Rohmer degradation (RD) in *Methylovulum psychrotolerans* strains Sph1^T^, OZ2, and Sph56 measured at 4, 10 and 20°C.

Strain		20°C	10°C	4°C
Av. 3 strains	C_30:1_	13 ± 4.6	10 ± 2.7	10 ± 2.1
	C_30:0_	19 ± 6.5	6.9 ± 3.3	7.2 ± 2.3
	C_31:1_	4.1 ± 17	3.1 ± 0.7	4.3 ± 1.6
	C_31:0_	18 ± 4.0	4.2 ± 1.1	9.5 ± 2.8
	C_32:1_	11 ± 1.1	45 ± 4.6	34 ± 7.8
	C_32:0_	36 ± 5.2	31 ± 1.9	34 ± 3.4
	Total C_30_	32 ± 10	17 ± 6.0	18 ± 4.2
	Total C_31_	22 ± 5.7	7.4 ± 1.4	14 ± 2.8
	Total C_32_	46 ± 5.0	76 ± 6.2	69 ± 5.2
	Total unsaturated	27 ± 4.1	58 ± 2.5	49 ± 7.7
	Ratio diplopterol/Σ RD hopanols	nd	0.1 ± 0.0	0.1 ± 0.0
Sph1^T^	C_30:1_	18 ± 0.8	10 ± 0.9	13 ± 1.2
	C_30:0_	22 ± 0.6	6.3 ± 0.5	8.5 ± 0.3
	C_31:1_	3.4 ± 0.0	2.7 ± 0.5	2.9 ± 0.3
	C_31:0_	16 ± 0.4	3.5 ± 1.1	8.6 ± 2.6
	C_32:1_	12 ± 0.2	47 ± 1.7	34 ± 3.6
	C_32:0_	30 ± 1.4	30 ± 1.3	34 ± 2.3
	Total C_30_	39 ± 1.4	16 ± 1.4	21 ± 1.0
	Total C_31_	19 ± 0.4	6.2 ± 1.6	12 ± 2.2
	Total C_32_	41 ± 0.9	77 ± 3.0	67 ± 1.3
	Total unsaturated	33 ± 0.9	60 ± 0.3	49 ± 5.1
	Ratio diplopterol/Σ RD hopanols	nd	0.1 ± 0.0	0.1 ± 0.0
OZ2	C_30:1_	13 ± 0.4	13 ± 0.4	11 ± 0.5
	C_30:0_	25 ± 0.3	11 ± 0.2	8.7 ± 0.1
	C_31:1_	2.8 ± 0.1	2.7 ± 0.1	3.8 ± 0.2
	C_31:0_	15 ± 0.3	5.1 ± 1.5	12 ± 0.1
	C_32:1_	9.2 ± 0.3	39 ± 1.0	26 ± 0.6
	C_32:0_	36 ± 0.5	29 ± 0.6	38 ± 0.2
	Total C_30_	38 ± 0.6	24 ± 0.1	19 ± 0.5
	Total C_31_	17 ± 0.2	7.8 ± 1.4	16 ± 0.1
	Total C_32_	45 ± 0.8	68 ± 1.6	64 ± 0.3
	Total unsaturated	25 ± 0.0	55 ± 0.7	41 ± 0.3
	Ratio diplopterol/Σ RD hopanols	nd	0.1 ± 0.0	0.1 ± 0.0
Sph56	C_30:1_	7.4 ± 0.3	7.2 ± 0.8	8.2 ± 1.3
	C_30:0_	11 ± 0.0	3.6 ± 0.4	4.3 ± 1.0
	C_31:1_	6.3 ± 0.5	3.9 ± 0.4	6.1 ± 1.4
	C_31:0_	23 ± 0.4	4.2 ± 0.4	7.4 ± 1.9
	C_32:1_	11 ± 0.1	48 ± 1.1	43 ± 3.7
	C_32:0_	41 ± 1.2	33 ± 1.0	31 ± 1.9
	Total C_30_	19 ± 0.4	11 ± 1.2	12 ± 2.3
	Total C_31_	29 ± 1.0	8.1 ± 0.8	13 ± 3.3
	Total C_32_	34 ± 0.4	53 ± 0.7	50 ± 1.8
	Total unsaturated	25 ± 0.8	60 ± 0.1	57 ± 0.9
	Ratio diplopterol/Σ RD hopanols	nd	0.1 ± 0.0	0.1 ± 0.0

### Changes in the Lipid Distribution With Changing Growth Temperature

**FIGURE 4:**
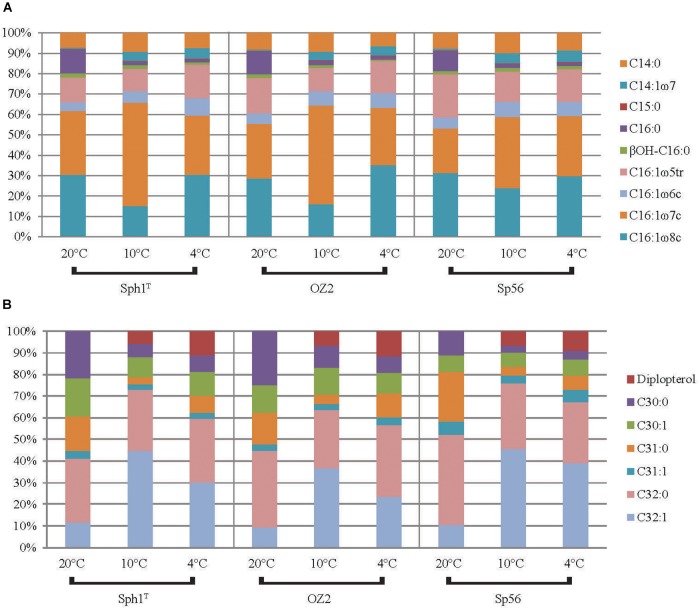
The distribution of **(A)** FAs and **(B)** hopanols formed by Rohmer degradation (including diplopterol) for the three *Methylovulum psychrotolerans* strains Sph1^T^, OZ2, and Sph56 (average of duplicate cultures) at the three growth temperatures.

The bulk-FA distributions of the three strains were so similar to each other at each temperature, that we discuss them further only as an average of the 6 cultures (three strains in duplicate). For the data of the individual strains see Table 4 and Figure 4. The majority of bulk-FA was C_16_ fatty acids. At the 20°C temperature optimum, 91 ± 0.5% of bulk-FAs were C_16,_ while at the two lower growth temperatures this percentage decreased slightly, to 86 ± 0.8 % at 10°C and 87 ± 1.5 % at 4°C. There was a concomitant increase in the C_14_ bulk-FA with decreasing temperature, from 8.3 ± 0.4% at 20°C, to 14 ± 0.7 % at 10°C and 13 ± 1.5 % at 4°C. The single C_15_ bulk-FA was a minor component and did not change with temperature (0.5 ± 0.0% at 20°C, 0.5 ± 0.1% at 10°C and 0.5 ± 0.1% at 4°C). The total percent of unsaturated bulk-FA (again an average of the 6 cultures, see Table 4 for data for the individual strains) increased with decreasing growth temperature. While it was 79 ± 2.0% at the 20°C optimum temperature, it had increased to 87 ± 3.1% at 10°C and 89 ± 0.9 at 4°C. The changes in total IPL-bound FAs with growth temperature were very similar to those of the bulk-FAs: the majority of fatty acids were C_16_ fatty acids, 82% at 20°C which decreased to 71% at 10°C and 70% at 4°C. As with the bulk-FAs there was a concomitant increase in the IPL-bound C_14_ fatty acids with decreasing temperature, from 16% at 20°C, to 26% at 10°C and 26% at 4°C. IPL-bound C_15_ fatty acids were a minor component and changed only slightly with temperature (2.0% at 20°C, 3.0% at 10°C and 3.5% at 4°C). The total percent of unsaturated IPL-bound FAs behaved similarly, to the bulk-FAs: it increased with decreasing growth temperature, from 69% at the 20°C to 80% at 10°C and 81 at 4°C.

**Table 4:** Relative percentage of bulk fatty acids in *Methylovulum psychrotolerans* strains Sph1^T^, OZ2, and Sph56 measured at 4, 10 and 20°C.

Strain		20°C	10°C	4°C
Av. 3 strains	Total C_16_	91 ± 0.5	86 ± 0.8	88 ± 1.5
	Total C_15_	0.5 ± 0.0	0.5 ± 0.1	0.5 ± 0.1
	Total C_14_	8.3 ± 0.4	14 ± 0.7	13 ± 1.5
	Total unsaturated	79 ± 2.0	87 ± 3.1	89 ± 0.9
	Ratio βOH-C_16:0_/ C_16:0_	0.2 ± 0.0	0.9 ± 0.2	0.9 ± 0.3
Sph1^T^	C_14:1ω7_	0.6 ± 0.1	4.5 ± 0.0	5.0 ± 0.1
	C_14:0_	7.2 ± 0.0	9.1 ± 0.1	7.4 ± 0.2
	C_15:0_	0.5 ± 0.0	0.5 ± 0.0	0.4 ± 0.0
	C_16:1ω8c_	31 ± 3.3	15 ± 1.9	30 ± 0.2
	C_16:1ω7c_	31 ± 3.2	51 ± 3.0	29 ± 0.2
	C_16:1ω6c_	4.3 ± 0.1	5.7 ± 0.2	8.5 ± 0.4
	C_16:1ω5tr_	12 ± 0.2	11 ± 0.9	16 ± 0.3
	C_16:0_	12 ± 0.1	1.7 ± 0.0	1.4 ± 0.0
	βOH-C_16:0_	1.9 ± 0.3	1.7 ± 0.2	1.2 ± 0.0
	Total C_16_	92 ± 0.1	86 ± 0.2	87 ± 0.1
	Total C_15_	0.5 ± 0.0	0.5 ± 0.0	0.4 ± 0.0
	Total C_14_	8.0 ± 0.0	14 ± 0.2	12 ± 0.1
	Total unsaturated	79 ± 0.2	87 ± 0.0	89 ± 0.2
	Ratio βOH-C_16:0_/ C_16:0_	0.2 ± 0.0	1.0 ± 0.1	0.9 ± 0.0
OZ2	C_14:1ω7_	0.7 ± 0.0	4.2 ± 0.0	4.5 ± 0.3
	C_14:0_	8.0 ± 0.0	9.1 ± 0.5	6.5 ± 0.2
	C_15:0_	0.6 ± 0.0	0.4 ± 0.1	0.5 ± 0.0
	C_16:1ω8c_	29 ± 0.0	16 ± 1.2	35 ± 0.1
	C_16:1ω7c_	27 ± 0.5	48 ± 0.8	28 ± 0.5
	C_16:1ω6c_	5.0 ± 0.0	7.0 ± 0.8	7.3 ± 0.1
	C_16:1ω5tr_	17 ± 0.3	12 ± 0.5	16 ± 0.1
	C_16:0_	11 ± 0.3	2.0 ± 0.1	1.4 ± 0.0
	βOH-C_16:0_	1.8 ± 0.1	1.3 ± 0.2	0.8 ± 0.0
	Total C_16_	91 ± 0.0	86 ± 0.6	88 ± 0.6
	Total C_15_	0.6 ± 0.0	0.4 ± 0.1	0.5 ± 0.0
	Total C_14_	9.0 ± 0.0	13 ± 0.5	11 ± 0.5
	Total unsaturated	79 ± 0.2	87 ± 0.9	91 ± 0.3
	Ratio βOH-C_16:0_/ C_16:0_	0.2 ± 0.0	0.6 ± 0.0	0.5 ± 0.0
Sph56	C_14:1ω7_	0.8 ± 0.0	4.9 ± 0.3	5.6 ± 0.2
	C_14:0_	7.7 ± 0.2	9.9 ± 0.3	8.5 ± 0.3
	C_15:0_	0.6 ± 0.1	0.6 ± 0.0	0.6 ± 0.0
	C_16:1ω8c_	31 ± 0.3	24 ± 1.8	30 ± 0.6
	C_16:1ω7c_	22 ± 0.0	35 ± 2.5	30 ± 0.3
	C_16:1ω6c_	5.6 ± 0.1	7.4 ± 0.1	7.1 ± 0.1
	C_16:1ω5tr_	21 ± 0.2	15 ± 0.3	16 ± 0.5
	C_16:0_	9.6 ± 0.3	1.7 ± 0.1	1.5 ± 0.1
	βOH-C_16:0_	1.7 ± 0.2	1.8 ± 0.2	1.7 ± 0.4
	Total C_16_	91 ± 0.2	85 ± 0.6	85 ± 0.5
	Total C_15_	0.6 ± 0.1	0.6 ± 0.0	0.6 ± 0.0
	Total C_14_	8 ± 0.1	15 ± 0.6	14 ± 0.4
	Total unsaturated	80 ± 0.6	86 ± 0.0	88 ± 0.7
	βOH-C_16:0_/ C_16:0_	0.2 ± 0.0	1.1 ± 0.0	1.2 ± 0.2

Previous studies have noted an increase of either more polar IPLs or more polar carotenoids in the membrane in adaption to decreased temperature for the psychrophilic *Clostridium psychromophilum* (Guan et al., 2013) and the psychrotolerant bacteria *Micrococcus roseus* (Chattopadhyay et al., 1997) and *Sphingobacterium antarcticus* (Jagannadham et al., 2000). However, in this study the IPL head group composition, and hence its polarity did not change with temperature: the ratio of PG to PE was 0.2 at all temperatures.

Another bulk-FA change with decreasing growth temperature was a relative increase in the β-hydroxy FA, βOH-C_16:0_. The ratio of βOH-C_16:0_ to C_16:0_ increased as growth temperature decreased, from 0.2 at 20°C, to 0.9 at both 10 and 4°C (Table 4). βOH-C_16:0_ has been detected in a range of methanotrophic bacteria (Bowman et al., 1991) and the source of many β-hydroxy FAs has been identified as the lipid A component of lipopolysaccharides (Wollenweber and Rietschel, 1990), which are a major constituent of the outer membrane of Gram-negative bacteria (Wollenweber and Rietschel, 1990; Keinänen et al., 2003). β-hydroxy FAs have been applied as biomarkers for Gram-negative bacteria in environmental studies (Klok et al., 1988; Wakeham et al., 2003; Lee et al., 2004; Wang et al., 2016) and the effect of temperature on the relative distribution of β-hydroxy FAs has been studied in the natural environment (Wang et al., 2016). Marr and Ingraham (1962) showed an increase in the relative percent of βOH-C_14:0_ in *Escherichia coli* from 6.9 to 12% as the growth temperature decreased from 25 to 10°C.

The majority of the hopanols at the temperature optimum of 20°C were C_32:0_ (36 ± 5.2%). The major change between the 20 and 10°C was that the relative percentage of C_32:1_ increased significantly, from 11 ± 1.1 % to 45 ± 4.6%. It was slightly lower at 4°C (34 ± 7.8%) but still significantly higher than at 20°C. This relative increase was countered by concomitant decreases in the other hopanols (Table 3). As was seen with the fatty acids, the percent of unsaturated hopanols increased for the cultures grown below the optimum growth temperature: from 27 ± 4.1% at 20°C, to 58 ± 2.5% at 10°C and 49 ± 7.7% at 4°C. The hopanols were also examined in terms of total C_30_ (saturated and unsaturated), C_31_ and C_32_. On average, the C_30_ sum decreased from 32 ± 10 at 20°C to 17 ± 6.0 at 10°C and 18 ± 4.2 at 4°C. The total C_31_ also decreased from 22 ± 5.7% at 20°C to 7.4 ± 1.4% at 10°C and 14 ± 2.8% at 4°C. Consequently, there was an increase in the total C_32,_ from 46 ± 5.0% at 20°C to 76 ± 6.2% at 10°C and 69 ± 5.2% at 4°C.

Overall, there was an increase in the percent of unsaturated hopanols as temperature decreased, along with a decrease in both the total C_30_ and C_31_ and concomitant increase in C_32_. In a mesocosm study of symbiotic methanotrophs in *Sphagnum* moss (van Winden, 2011; van Winden et al., 2012a), no unsaturated BHPs were reported, however, the relative percentage of aminotriol BHP (corresponding to C_32_ hopanol) increased as temperature decreased (from 25 to 5°C), while both aminotetrol BHP (corresponding to C_31_ hopanol) and aminopentol BHP (corresponding to C_30_ hopanol) decreased. Similarly, Osborne et al. (2017) also reported changes in the BHP distribution with temperature in methane-amended aerobic river-sediment incubations. In those temperature treatments relevant to this study, it was shown that with decreasing temperature (from 40°C to 4°C) there was also an increase in the relative percentage of aminotriol BHP (corresponding to C_32_ hopanol), while aminopentol BHP decreased and aminotetrol BHP remained constant (Osborne et al., 2017). In contrast, Jahnke et al. (1999) reported that for a psychrotolerant methanotroph (thought to be *Methylomonas methanica*) the relative percentage of C_32_ hopanol remained the same as temperature decreased, while the C_30_ hopanol increased and the C_31_ hopanol decreased. Overall, based on our results and the limited reports in the literature, it would seem that methanotrophic bacteria adapt their BHP distribution to adapt to colder temperatures, by increasing the relative amount of tetrafunctionalised BHPs (either in the saturated or unsaturated form).

In all three *M. psychrotolerans* strains diplopterol was not detected in the cultures grown at 20°C but the ratio of diplopterol to the sum of the six hopanols was on average at 0.1 ± 0.0 at both 10 and 4°C. During hopanoid biosynthesis, the cyclisation of squalene results in either the production of diploptene or diplopterol, but it is diploptene that is reported to be the pre-cursor in the biosynthesis of BHPs. The relative increase in diplopterol with decreasing temperature suggests an additional membrane adaption separate from that of the BHPs.

### Processes and Implications of Lipid Remodeling

**FIGURE 5:**
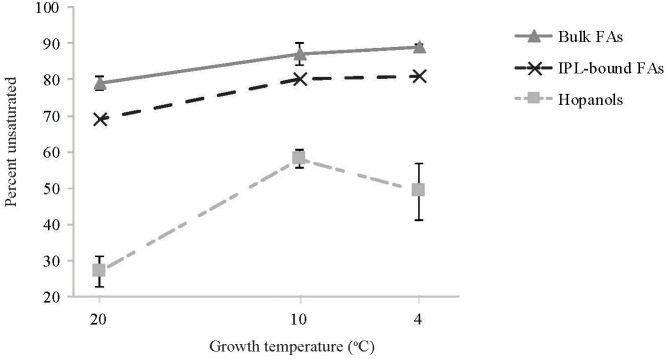
The change with temperature in the percent of unsaturated lipids for different lipid classes: bulk fatty acids (FAs), intact polar lipid (IPL)-bound FAs, hopanols (formed by Rohmer degradation). For bulk FAs and hopanols the data represents the avarage of strains Sph1^T^, OZ2 and Sph56 all in dulplicate cultures. For IPL-bound FAs the data represents single replicate cultures of strain Sph56.

Overall, as the growth temperature decreased from 20 to 4°C, the percent of unsaturated *M. psychrotolerans* bulk-FAs increased from 79 to 89% while the total percent of unsaturated IPL-bound-FAs increased from 69 to 81% (Figure 5). Increased FA unsaturation in response to decreased temperature is a commonly observed response in order to maintain the liquid-crystalline character of bacterial membranes (Marr and Ingraham, 1962; Suutari and Laakso, 1994; Chattopadhyay, 2006; Guschina and Harwood, 2006; Barria et al., 2013; Siliakus et al., 2017). Certain bacteria have been shown to contain cold acclimation proteins (Caps) (Jones and Inouye, 1994; Berger et al., 1996), which include acyl lipid desaturases (Carty et al., 1999; Vorachek-Warren et al., 2002; Albanesi et al., 2004). These enzymes belong to the FA desaturase family (FAD) and are known to be regulated in all organisms by feedback mechanisms in which sensor proteins control the transcription of genes that modify pre-formed lipids (Aguilar and de Mendoza, 2006).

Hopanoids are involved in increasing membrane stability in certain bacteria (Welander et al., 2009; Schmerk et al., 2011) and their structure can undergo post-synthesis modifications such as methylation and side chain modifications (Welander and Summons, 2012). The most notable hopanoid temperature adaption of *M. psychrotolerans* was an increase in unsaturated hopanoids with decreasing temperature, from 27 to 49% (Figure 5). The presence of hopanoids is a common feature to all investigated obligate methanotrophs (Cvejic et al., 2000b), however very few studies have described the presence of unsaturated hopanoids. Furthermore, increased hopanoid unsaturation upon cold exposure has not been described previously and hence the enzymes responsible for the desaturation of the hopanoid core have not previously been established.

In order to investigate the mechanisms of both FA and hopanoid cold-adaption in *M. psychrotolerans*, we examined its genome for *M. psychrotolerans* for the presence of genes that potentially code for fatty acid desaturases. We identified the AADEFJLK_03363 gene that codes for the POZ50891 protein, which is annotated as a potential FA desaturase enzyme. Topology analysis of this protein predicted that it forms four trans-membrane helices, which suggests it is an integral membrane protein (Aguilar et al., 1998). In addition, enzymes from the FAD usually contain highly conserved histidine-rich motifs, which we also identified in POZ50891 FA desaturase (H_98_XXXH_102_, H_134_XXH_137_H_138_, H_261_H_262_XH_264_, H_307_XXH_310_H_311_). In order to investigate the enzymatic mechanisms responsible for hopanoid unsaturation, we searched for genes that potentially code for proteins involved in this reaction. We identified a gene AADEFJLK_00554 coding for a potential sterol desaturase POZ53527 protein, belonging to the FA hydroxylase (FAH) superfamily of proteins. Within this group, a wide set of enzymatic activities related to hydroxylation, desaturation and oxidation have been described (Arthington et al., 1991; Bard et al., 1996; Mitchell and Martin, 1997; Ternes et al., 2002; Vences-Guzmán et al., 2011) (cf. Supplementary Table S3). Sterol and sterol-derived molecules are not produced by *M. psychrotolerans*, but hopanoids are considered structural analogs, therefore we postulate that the POZ53527 protein present in *M. psychrotolerans* could be involved in hopanoid desaturation, as has been postulated previously for similar sterol desaturases (Tushar et al., 2014). Topology analysis of the POZ53527 protein revealed five trans-membrane helices, suggesting that this protein is also an integral membrane protein, as well as the histidine rich motifs typically found in FAH family of proteins (Supplementary Figure S3).

Both the FA desaturase and the sterol desaturase identified here are integral membrane histidine motif-containing enzymes (IMHME) (Cid et al., 2017). Their sequence analyses revealed that they belong to the FAD and the FAH superfamilies, respectively. According to this classification, *M. psychrotolerans* contains one protein of each group, and both proteins can be actively involved in lipid modification under low temperature conditions. Among the FAD family of proteins, the bacterial FA desaturase (Δ5-Des) of *B. subtilis* has been characterized and it is known to be transcriptionally regulated in response to decreased temperature (Aguilar et al., 1998). Proteins that belong to the FAH family of proteins, are very diverse in their activities and substrate specificities (Cid et al., 2017) (Summarized in Supplementary Table S3). The potential *M. psychrotolerans* hopanoid desaturase protein POZ53527 belongs to this classification and will represent a novel enzymatic activity related to the substrate specificity to hopanoids. Thus, we propose that the unsaturation of hopanoids represents a novel membrane remodeling feature in order to maintain the membrane homeostasis upon cold adaptation. If this hypothesis is correct, these results may be useful for studies of the natural environment. Indeed, it may possible to make inferences from the proportion of Δ^11^ unsaturated hopanoids about growth temperature or the ecological niche of BHP producers, in particular in low-temperature environments. However, to date the reports of Δ^11^ unsaturated hopanoids in the natural environment are limited (e.g., Cooke et al., 2008; Handley et al., 2010; Spencer-Jones et al., 2015; Rush et al., 2016).

## Conclusion

Three strains of *Methylovulum psychrotolerans* were grown at three different growth temperatures, 20°C, 10°C and 4°C and were capable of oxidizing methane down to 4°C. The three *M. psychrotolerans* strains adapted to decreasing growth temperature by increasing unsaturation in both FAs (bulk and IPL-bound) and hopanoids. The bulk FA ratio of βOH-C_16:0_ to C_16:0_ increased as growth temperature decreased. The hopanol composition of *M. psychrotolerans* contains C_30:0,_ C_31:0_ and C_32:0_ along with their unsaturated forms. The most notable hopanoid adaption *M. psychrotolerans* exhibited to lower growth temperatures was an increase in C_32:1_ hopanols (formed from unsaturated tetrafunctionalised BHPs). The total sum of C_32_ hopanols increased as temperature decreased. Similar results have been reported in other studies. Diplopterol was not detected in the cultures grown at 20°C but was present at 10 and 4°C.

Our results demonstrate that not only FAs, but also hopanoids can be remodeled to maintain bacterial membrane homeostasis upon cold adaptation. We identified genes that potentially code for FA lipid desaturases and for hopanoid unsaturation in the genome of *M. psychrotolerans*. Further work would be needed to produce the biochemical evidence of the enzymatic activity of the proposed membrane-adaption genes in *M. psychrotolerans*.

## Author Contributions

NB carried out data analysis and wrote the manuscript. WR carried out extractions and lipid analysis. DS-C carried out genomic analyses. IO and SB cultured bacteria and measured methane oxidation rates. SD and JSD designed and supervised the study.

## Conflict of Interest Statement

The authors declare that the research was conducted in the absence of any commercial or financial relationships that could be construed as a potential conflict of interest.
