# Supplementary material for: Fatty Acid and Hopanoid Adaption to Cold in the Methanotroph Methylovulum psychrotolerans
Source: Front Microbiol. 2019 Apr 5;10:589. doi: 10.3389/fmicb.2019.00589 (PMC6460317; doi:10.3389/fmicb.2019.00589)
Supplement: Supplementary file 1 [file Data_Sheet_1.docx]

**Supplement to: Fatty acid and hopanoid adaption to cold in the** **psychrotolerant methanotroph *Methylovulum psychrotolerans***

Nicole J. Bale^1*^, W. Irene C. Rijpstra^1^, Diana X. Sahonero-Canavesi^1^, Igor Y. Oshkin^2^, Svetlana E. Belova^2^, Svetlana N. Dedysh^2^, Jaap S. Sinninghe Damsté^1,3^

^1^Department of Marine Microbiology and Biogeochemistry, NIOZ Royal Institute for Sea Research, and Utrecht University, Den Burg, Texel, The Netherlands

^2^Research Center of Biotechnology of the Russian Academy of Sciences, Winogradsky Institute of Microbiology, Moscow, Russia.

^3^Department of Earth Sciences, Faculty of Geosciences, Utrecht University, Utrecht, the Netherlands.

**Table S1.** Growth rates (h^-1^) of *Methylovulum psychrotolerans* strains Sph1^T^, OZ2, and Sph56 at 4, 10 and 20^o^C.

| **Strain** | **Temperature (°C)** | | |
| --- | --- | --- | --- |
|  | **20** | **10** | **4** |
| **Sph1^T^** | 0.061 | 0.038 | 0.013 |
| **OZ2** | 0.064 | 0.031 | 0.016 |
| **Sph56** | 0.057 | 0.041 | 0.012 |

**Table S2.** Doubling time of *Methylovulum psychrotolerans* strains Sph1^T^, OZ2, and Sph56 cultivated at 4, 10 and 20^o^C.

| **Strain** | **Time (h)** | | |
| --- | --- | --- | --- |
|  | **20** | **10** | **4** |
| **Sph1^T^** | 11.4 | 18.3 | 53.7 |
| **OZ2** | 10.8 | 22.6 | 44.2 |
| **Sph56** | 12.1 | 16.9 | 56.1 |

**Table S3.** Potential and characterized members of the FAH superfamily involved in different enzymatic activities: sterol desaturase, sterol deethylase, ornithine lipid hydroxylase, C-4 sterol methyl oxidase, ceramide very long chain fatty acid and very-long-chain aldehyde decarbonylase. Amino acid sequences were analyzed in the PFAM database (El-Gebali et al.), and presence of transmembrane Helix (TMH) was analyzed with OCTOPUS (Viklund and Elofsson, 2008).

| **Accession** | **Protein name (Uniprot)** | **Gene** | **PFAM** | **TMH** | **Reference** | **Enzymatic activity** | **Organism** |
| --- | --- | --- | --- | --- | --- | --- | --- |
| POZ53527.1 | Sterol desaturase | AADEFJLK_00554 | PF04116 | 5 | This work | Hopanoid desaturase | *Methylovulum psychrotolerans Sph1^T^* |
| P32353 | Delta(7)-sterol 5(6)-desaturase | ERG3 | PF04116 | 4 | (Arthington et al., 1991) | Ergosterol desaturase | *Saccharomyces cerevisiae ATCC 204508* |
| XP_001017777 | Sterol desaturase | TTHERM_00438800 | [PF04116](http://pfam.xfam.org/family/PF04116) | 4 | (Tomazic et al., 2011) | Sterol deethylase | *Tetrahymena thermophila SB210* |
| WP_015340785 | Ornithine lipid hydroxylase | olsE | PF04116 | 5 | (Vences-Guzmán et al., 2011) | Hydroxylation of ornithine head group | *Rhizobium tropici CIAT 899* |
| P53045 | Methylsterol monooxygenase | ERG25 | PF04116 | 4 | (Bard et al., 1996) | C4 methyl oxidase | *Saccharomyces cerevisiae ATCC 204508* |
| Q03529 | Ceramide very long chain fatty acid hydroxylase SCS7 | SCS7 | PF04116 / PF00173 | 4 | (Mitchell and Martin, 1997) | Hydroxylation of very long chain fatty acid of dihydroceramides at C-2 | *Saccharomyces cerevisiae ATCC 204508* |
| F4HVY0 | Very-long-chain aldehyde decarbonylase CER1 | CER1 | PF04116 / PF12076 | 6 | (Aarts et al., 1995) | Decarbonylase | *Arabidopsis thaliana* |

**Figure S1.** Geographic locations of the three sampling sites, where strains Sph1^T^ (1), OZ2 (2) and Sph56 (3) were isolated.


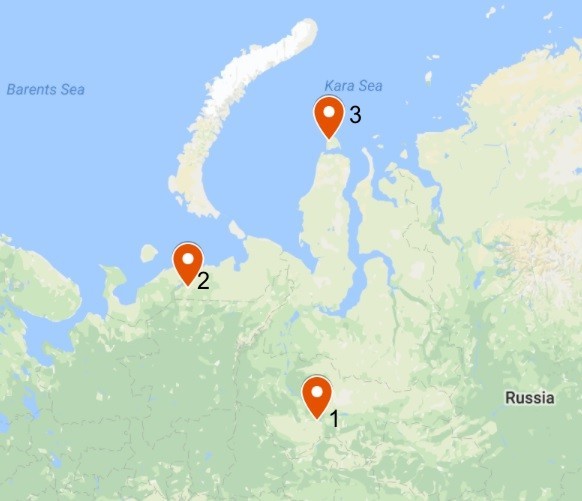


**Figure S2.** Growth dynamics of strains Sph1^T^ (A), OZ2 (B), and Sph56 (C) at different incubation temperatures. Circles, triangles and squares indicate incubations at 20, 10 and 4^o^C, respectively. Data are means of triplicate.


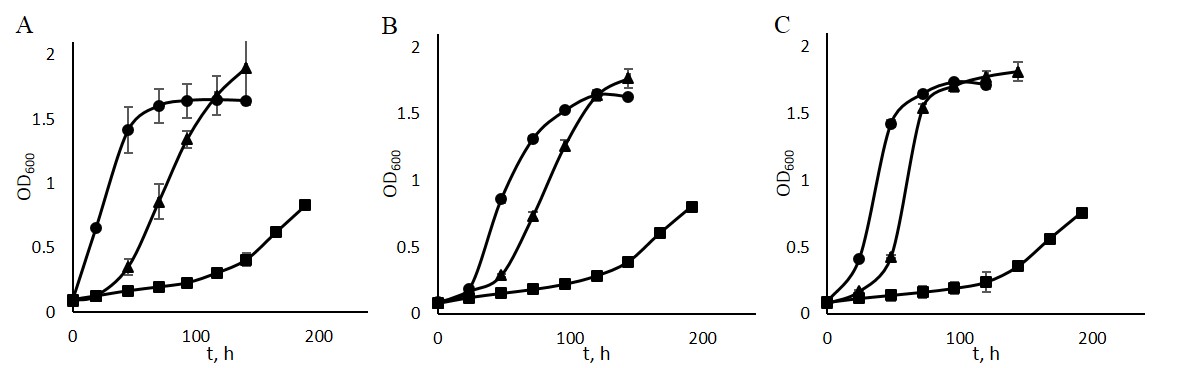


**Fig. S3** Multiple Alignment of histidine rich regions of *M. psychrotolerans* potential hopanoid desaturase POZ53527.1 compared with other characterized members of the fatty acid hydroxylase (FAH) superfamily. Proteins that belong to the FAH superfamily contain two copies of a HXHH motif. Sequences used for the alignment were the potential hopanoid desaturase; POZ53527.1 (*M. psychrotolerans sph1*), sterol desaturase; P32353 (*Saccharomyces cerevisiae* ATCC 20450), sterol deethylase; XP_001017777 (*Tetrahymena thermophila* SB210), ornithine lipid hydroxylase; WP_015340785 (*R. tropici* CIAT 899), C-4 sterol methyl oxidase; P53045 (*S. cerevisiae strain* ATCC 204508), ceramide very long chain fatty acid hydroxylase; Q03529 (*S. cerevisiae* ATCC 20450) and very-long-chain aldehyde decarbonylase; F4HVY0 (*A. thaliana*).

POZ53527.1_M.psychrotolerans 92 YWQHV-ASH 99 104 LWR-LHQVHHS 113 188 LHRIHHS 194

P32353_S.cerevisiae 197 YLAHR-WLH 204 208 VYRALHKPHHK 218 287 CHTVHHL 293

XP_001017777_T.thermophila 141 YWKHR-LLH 148 152 FWY-FHRNHHS 161 240 HHNLHHS 246 WP_015340785_R.tropici 138 YMAHR-LAH 145 150 LWR-FHALHHS 159 231 LHRWHHS 237

P53045_S.cerevisiae 157 YWAHR-LFH 164 168 FYKYIHKQHHR 178 263 HHDLHHH 269

Q03529_S.cerevisiae 241 YGLHRFLFH 249 264 HFL-LHGCHHY 273 344 YHLEHHY 350

F4HVY0_A.thaliana 143 YWLHK-ALH 150 154 LYSRYHSHHHS 164 247 YHSLHHT 253

Consensus * * * ** * **

**References**

Aarts, M. G., Keijzer, C. J., Stiekema, W. J., and Pereira, A. (1995). Molecular characterization of the CER1 gene of arabidopsis involved in epicuticular wax biosynthesis and pollen fertility. *The Plant Cell* 7, 2115–2127. doi:10.1105/tpc.7.12.2115.

Arthington, B. A., Bennett, L. G., Skatrud, P. L., Guynn, C. J., Barbuch, R. J., Ulbright, C. E., et al. (1991). Cloning, disruption and sequence of the gene encoding yeast C-5 sterol desaturase. *Gene* 102, 39–44.

Bard, M., Bruner, D. A., Pierson, C. A., Lees, N. D., Biermann, B., Frye, L., et al. (1996). Cloning and characterization of ERG25, the Saccharomyces cerevisiae gene encoding C-4 sterol methyl oxidase. *Proc. Natl. Acad. Sci. U.S.A.* 93, 186–190.

El-Gebali, S., Mistry, J., Bateman, A., Eddy, S. R., Luciani, A., Potter, S. C., et al. The Pfam protein families database in 2019. *Nucleic Acids Res*. doi:10.1093/nar/gky995.

Mitchell, A. G., and Martin, C. E. (1997). Fah1p, a Saccharomyces cerevisiae Cytochromeb 5 Fusion Protein, and ItsArabidopsis thaliana Homolog That Lacks the Cytochromeb 5 Domain Both Function in the α-Hydroxylation of Sphingolipid-associated Very Long Chain Fatty Acids. *J. Biol. Chem.* 272, 28281–28288. doi:10.1074/jbc.272.45.28281.

Tomazic, M. L., Najle, S. R., Nusblat, A. D., Uttaro, A. D., and Nudel, C. B. (2011). A Novel Sterol Desaturase-Like Protein Promoting Dealkylation of Phytosterols in Tetrahymena thermophila▿. *Eukaryot Cell* 10, 423–434. doi:10.1128/EC.00259-10.

Vences-Guzmán, M. Á., Guan, Z., Ormeño-Orrillo, E., González-Silva, N., López-Lara, I. M., Martínez-Romero, E., et al. (2011). Hydroxylated ornithine lipids increase stress tolerance in Rhizobium tropici CIAT899. *Mol. Microbiol.* 79, 1496–1514. doi:10.1111/j.1365-2958.2011.07535.x.

Viklund, H., and Elofsson, A. (2008). OCTOPUS: improving topology prediction by two-track ANN-based preference scores and an extended topological grammar. *Bioinformatics* 24, 1662–1668. doi:10.1093/bioinformatics/btn221.
